# Cellular interaction of mycosis fungoides tumor cells changes from cytotoxic CD8+ T cells in plaques to B cells in tumors

**DOI:** 10.3389/fimmu.2026.1823626

**Published:** 2026-07-02

**Authors:** Veerle A. Merkus, Marieke E. IJsselsteijn, Marie S. N. Chevalier, Sanne de Haan, Vincent van Unen, Cornelis P. Tensen, Abdoel El Ghalbzouri, Anne M. R. Schrader, Ferenc A. Scheeren, Noel F. C. C. de Miranda, Maarten H. Vermeer, Koen D. Quint

**Affiliations:** 1 Department of Dermatology, Leiden University Medical Centre, Leiden, Netherlands; 2 Department of Pathology, Leiden University Medical Centre, Leiden, Netherlands; 3 Department of Immunology, Leiden University Medical Centre, Leiden, Netherlands

**Keywords:** cancer immunology, cutaneous T cell lymphoma, imaging mass cytometry, immune landscape, mycosis fungoides, spatial, spatial analyses, tumor microenvironment

## Abstract

**Introduction:**

Mycosis Fungoides (MF) is characterized by the proliferation of malignant skin-homing memory CD4+ T cells. The disease typically follows an indolent course, progressing from plaques (scaly, erythematous skin lesions) in the early stages (Ia-Ib) to tumors (>IIb) in approximately one-third of cases. Although recent studies have explored cell phenotypes and counts of the tumor microenvironment (TME) in MF progression, spatial interactions between tumor and reactive cells remain poorly understood.

**Methods:**

We performed a comprehensive high-dimensional analysis of immune cell composition and cellular interactions across MF stages using a custom Imaging Mass Cytometry (IMC) panel to examine the spatial complexity of the MF TME.

**Results:**

Stage-specific changes in the TME included an increased percentage of cytotoxic CD8+ cells in plaques and an increased percentage of B cells and dendritic cells in tumors. With progression of disease shift in the spatial organization of the TME was observed, from CD8+ T cell–tumor cell and monocyte–CD4+ T cell interactions in plaques to B cell–tumor cell interactions in tumors.

**Discussion:**

A stage-dependent shift in interactions from anti-tumor immune responses to features consistent with immune evasion mechanisms has been observed with the progression of MF. These insights underscore the importance of spatial context in understanding MF progression and highlight therapeutic targets that could inform stage-specific (immune)therapies.

## Graphical Abstract

**Figure:**
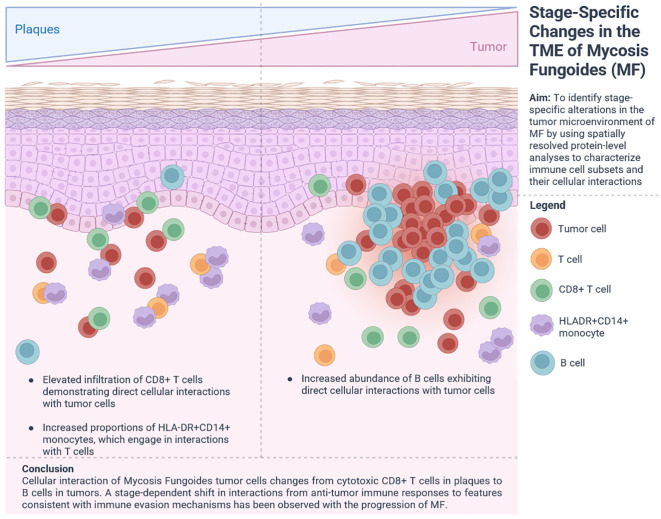

## Introduction

Mycosis Fungoides (MF) is the most common form of primary cutaneous T-cell lymphoma (CTCL), typically characterized by proliferation of malignant T cells derived from skin-homing memory CD4+ T cells (1, 2). The disease typically follows a slow progressive course, presenting in the early stages (stages I–IIA) with erythematous patches and plaques that can be treated with skin-directed therapies including topical steroids, chlormethine gel, and PUVA/UVB light therapy (3). In approximately one-third of cases, the disease progresses to an advanced stage (stage ≥ IIB), characterized by tumor formation and associated with a low overall survival rate (2, 4, 5). Patients with advanced stage disease are commonly treated with radiotherapy combined with systemic therapies, such as retinoids, interferons, photopheresis, HDAC inhibitors, or single-agent chemotherapy; however, response rates remain low (20–35%) and typically last only 4–6 months (6). Recently, the characterization of malignant T cells has led to new treatment options, including mogamulizumab (a monoclonal antibody against CCR4) and brentuximab vedotin (an antibody-drug conjugate against CD30) (7–9).

Despite these advances, treatment remains largely noncurative, highlighting the need for deeper insight into disease mechanisms to improve patient outcomes. Previous studies have investigated the correlation between genetic and epigenetic changes in tumor cells and disease progression (10). More recent research has focused on changes in the tumor microenvironment (TME) and their association with disease progression (11). With disease progression, the percentage of infiltrating reactive CD8+ T cells declines, whereas the number of B cells increases (12, 13). In a single-cell RNA sequencing dataset, putative ligand-receptor interactions between tumor cells and B cells via CD28-CD86 and CD70-CD27 were identified in advanced stage MF (13). This is accompanied by a shift from a Th1-type immune response to a Th2-dominant response that supports tumor proliferation and immune evasion (14–16). Furthermore, MF progression has been associated with tumor-associated M2-like macrophages (17, 18). Overall, early stage MF appears to exhibit an immune environment that constrains tumor growth, whereas advanced stage MF is marked by immune-suppressive changes that support tumor proliferation and immune evasion (14).

Although valuable insights into specific immune cell populations and predicted cellular interactions have been gained from ligand–receptor analyses based on RNA expression, the spatial organization of these cell types at the protein level remains insufficiently understood. In particular, quantifying and identifying different myeloid subsets, including monocytes and dendritic cells, and defining their spatial arrangement are essential for clarifying the immune dynamics of MF. A more comprehensive, spatially resolved approach can examine the tumor microenvironment, enabling precise identification of immune cell subsets and deeper insight into MF−associated immune behavior. This has become increasingly relevant in light of emerging therapeutic implications and the opportunity to guide the development of improved and more precise treatment strategies.

To address these challenges, we used Imaging Mass Cytometry (IMC), a high-dimensional imaging technique that combines spatial resolution with mass spectrometry’ multiplexing. IMC enables simultaneous detection of numerous protein markers, revealing cell types, states, and cellular interactions within the TME (19). We developed an MF-specific IMC panel that included key immune, stromal, and tumor markers, allowing for the analysis of over 40 protein markers in a single tissue section. Specifically designed and optimized for formalin fixed paraffin-embedded (FFPE) skin biopsies, this panel offers insights into MF, with a focus on both patches/plaques and tumors. Our spatial analysis provides a comprehensive view of the immune landscape including T cell and myeloid subset identification, and cellular interactions across early and advanced stages of MF, providing a deeper understanding of tumor progression and the TME.

## Materials and methods

### Patient material selection

A total of 27 skin biopsies (4mm) from 20 patients with confirmed diagnoses of classical CD4+ MF (stages IA-IV) were selected from the Biobank Skin Diseases of the Leiden University Medical Center. Eight patch/plaque biopsies from early-stage patients and 11 tumor biopsies from late-stage patients were included. Additionally, biopsies from 4 late-stage patients with both plaques and tumors were included, with plaque and tumor biopsies taken at the same time. All biopsies were formalin fixed and paraffin embedded (FFPE). Diagnoses followed the WHO-EORTC criteria (2) and was conducted by an expert panel of dermatologists and pathologists from the Dutch Cutaneous Lymphoma Working Group (DCLWG). Furthermore, all early stage patients had stable disease for at least two years prior to biopsy. All the patients provided written informed consent for biobanking. The study was approved by the Institutional Review Board (protocol: B19.005) and adhere to the ethical guidelines and the Declaration of Helsinki. The baseline characteristics and disease course are shown in **Table 1**.

**Table 1:** Patient characteristics.

Characteristic	Early stage (1a-1b)	Advanced stage (2b-4)
No. of patients	8	12
Biopsies		
Plaques	8	4
Tumors	0	15
Age, years		
Median	55	76
Range	16-80	40-78
Sex		
Male	6 (75%)	8 (67%)
Female	2 (25%)	4 (33%)
Current status		
Alive without disease	0	0
Alive with disease	7	8
Died of unrelated disease	0	0
Died of lymphoma	1*	4

*At the time of diagnosis patient had early-stage MF. 5 years after plaque biopsy advanced stage developed.

### Panel optimization and antibody conjugation

Antibody selection was based on the literature and a panel pre-tested by Ijsselsteijn et al. (20). The antibodies were first tested using immunohistochemistry (IHC). Antibodies that demonstrated strong IHC performance were subsequently conjugated to a heavy metal and evaluated using the Hyperion Imaging System (Standard BioTools, San Francisco, CA, USA). The antibodies were conjugated in-house with heavy metal isotopes using the MaxPar X8 Polymere Antibody Labeling Kit, according to the manufacturer’s protocol (Standard BioTools, California, USA). The conjugation of anti-α-SMA to 209Bi was performed using a protocol from Krop et al. (21, 22). Conjugation of 194Pt, 196Pt, and 198Pt (Standard BioTools, California, USA) to respectively anti-vimentin, anti-SPARC, and anti-keratin was performed as described by Mei et al. (23) The antibodies were tested at different dilutions (1:50, 1:100, 1:200) and staining conditions (4 °C overnight (ON) or 5 hours Room Temperature (RT)). The optimal dilution and staining condition were selected based on the images acquired using the Hyperion Imaging System (Standard BioTools, San Francisco, CA, USA). The panel with heavy metal isotope-tagged monoclonal antibodies are listed in **Table 2** and visible in **Supplementary Figure 1**.

**Table 2:** Imaging mass cytometry antibody panel.

Target	Clone	Metal	Time	Temperature	Dilution	RRID
CD90	D3v8A	89y	Overnight	4 °C	100	AB_3076750
CD31	89C2	115Ln	Overnight	4 °C	100	AB_2160882
HLA-DR	TAL 1B5	141Pr	5 h	RT	100	AB_1017909
CD20	H1	142Nd	Overnight	4 °C	100	AB_396030
CD68	D4B9C	143Nd	Overnight	4 °C	100	AB_2799882
CD11b	D6X1N	144Nd	5 h	RT	100	AB_2799357
CD4*	EPR6855	145Nd	Indirect ON	4 °C	100	AB_2750883
CD8a	D8A8Y	146Nd	5 h	RT	50	AB_2800052
TIGIT	BLR047F	147Sm	5 h	RT	100	AB_2943164
CCR6	MM0066-3L1	148Nd	Overnight	4 °C	100	AB_3256375
Collagen 1	EPR7785	149Sm	Overnight	4 °C	100	AB_2909621
Granzyme B	D6E9W	150Nd	5 h	RT	100	AB_2799313
CD66b	G10F5	151Eu	5 h	RT	50	AB_314494
Ki-67	8D5	152Sm	Overnight	4 °C	100	AB_2797703
CD3	EP449E	153Eu	Overnight	4 °C	50	AB_3698033
TCRyd**	H41	154Sm	Indirect ON	4 °C	100	AB_1130061
CD141	E7Y9P	155Gd	Overnight	4 °C	50	AB_3718004
PD-L1	E1L3N(R)	156Gd	Overnight	4 °C	50	AB_2922774
IDO	D5J4E(TM)	157Gd	Overnight	4 °C	100	AB_3683091
CD1c	EPR23189-196	158Gd	5 h	RT	50	AB_2884015
FoxP3	D608R	159Tb	Overnight	4 °C	50	AB_3713188
PD-1	D4W2J	160Gd	5 h	RT	50	AB_3675993
ICOS	D1K2T(tm)	161Dy	5 h	RT	50	AB_3676096
CD207	310F7.02	162Dy	5 h	RT	50	AB_3107495
CD14	D7A2T	163Dy	5 h	RT	100	AB_2799504
CD47	B6H12	164Dy	5 h	RT	50	AB_2865739
CD45RO	UCHL1	165Ho	Overnight	4 °C	100	AB_2799491
CD30	BerH2	166Er	5 h	RT	50	AB_627023
CD56	E7X9M	167Er	5 h	RT	100	AB_3674471
CD103	EPR4166(2)	168Er	5 h	RT	50	AB_3697252
TCRβ1	E6Z3S	169Tm	5 h	RT	50	AB_3675279
CD45RA	Hi100	170Er	5 h	RT	100	AB_2562822
CD15	MC480	171Yb	Overnight	4 °C	100	AB_1264258
CD1a	EP3622	172Yb	Overnight	4 °C	100	AB_1267034
CD163	D6U1J	173Yb	5 h	RT	50	AB_3675763
CD7	EPR4242	174Yb	5 h	RT	100	AB_2889384
CD45	D9M8I	175Lu	Overnight	4 °C	50	AB_2922773
CD11c	EP1347Y	176Yb	5 h	RT	100	AB_2864379
DNA	NA	191-193lr	5 min	RT	100	
Vimentin	D21H3	194Pt	Overnight	4 °C	50	AB_3679405
SPARC	AF941	196Pt	Overnight	4 °C	50	AB_2302498
Keratin	C11 and AE1/AE3	198Pt	Overnight	4 °C	50	AB_439775
αSMA	D4K9N	209bi	5 h	RT	100	AB_3662123

RT, room temperature.

*Secondary antibody anti-rabbit (AB_272685) conjugated to ^145^Nd was used for staining.

**Secondary antibody anti-mouse (AB_956005)conjugated to ^155^Gd was used for staining.

### Imaging mass cytometry staining and data acquisition

FFPE blocks with MF skin tissues were cut twice into 4 μm sections and placed on two silane-coated glass slides (VWR, Radnor, PA, USA). One slide was used for Hematoxylin and Eosin (HE) staining, and the consecutive slide was used for IMC staining. Procedures for IMC antibody staining and data acquisition for FFPE tissues were performed as previously described (20). IMC antibody staining was performed according to an optimized protocol, as previously stated, and the staining conditions are shown in **Table 2**. IMC images were acquired using the Hyperion Imaging System (Standard BioTools, San Francisco, CA, USA). All acquisitions were performed in accordance with the manufacturer’s protocol. After daily calibration, 1mmx1mm Regions of interest (ROI) were selected based on the consecutive HE slides. Within the HE slide, two regions of 1mmx1mm were selected, usually covering the whole epidermis. Within the Hyperion, corresponding ROIs can be identified based on a panoramic photograph of the slide, depicting the tissue structure. The tissue slides were subjected to laser ablation at a resolution of 1 µm and frequency of 200 Hz. All IMC data were stored as MCD and txt files.

### IMC image processing, cell segmentation and cell clustering analyses

All images and markers were checked for quality using the MCDViewer (Standard Biotools, version 1.0.560.6). Images were exported as 16-bit multi-TIF files and imported into Jupyter notebook with PENGUIN. PENGUIN was used for background removal while preserving the signal intensity differences and reducing noise (24). Subsequently, the cells were segmented using Cell Profiler (version 4.2.6). Single-cell protein expression was used for cell-type identification. Cell types were identified in three steps via dimensionality reduction to visualize high-dimensional data using Cytosplore (version 2.3.1). First, h-SNE was performed using 15 markers. Three major cell type groups were clustered (myeloid, lymphoid, and other cells) based on the protein expression pattern of each cluster. Subsequently, the major cell type groups were separately clustered in a t-SNE to identify the cell subsets (**Supplementary Figure 2B**). Per major cell type group, markers of interest were selected to identify the phenotypes. For the myeloid population mostly myeloid markers were added to the t-SNE (CD141, CD56, CD68, CD163, HLA-DR, CD66b, CD1a, CD11c, CD14, CD207, CD11b, CD45, CD45RO, CD1c). For the lymphoid population CD56, CD30, CD45, CD45RO, CD45RA, CD4, CD103, CD3, TCRb1, CD20, CD7, CD8a, FoxP3, TCRyd were added to the t-SNE. For other cells CD90, Vimentin, CD68, HLA-DR, Collagen1, CD31, CD66b, CD45, Keratin, a-SMA, CD3 were added to the T-SNE. To ensure proper cell type identification, for example T cell markers were added to the “other cell types” group as well to validate that there were no T cells left in that group. Finally, CD4+ T cells were separately clustered in a t-SNE to identify the CD4+ tumor cells. For this clustering the following markers were used: CD45RO, CD45RA, CD45, CD30, CD4, CD8a, TCRyd, FoxP3, CD103, TCRb1, CD7 and Ki67. In total, 24 phenotypes were identified in myeloid, lymphoid, and stromal cell populations using dimensionality reduction t-SNE. Phenotypes were loaded back into ImacytE (25) to verify the phenotype location and protein marker expression. Further analyses was conducted in R (v.4.3.2), including cell quantification, statistical analyses, cellular percentages and spatial cellular interaction analyses. The Mann-Whitney unpaired nonparametric t-test was used as a statistical test to compare cell counts and cell percentages between plaques and tumors. Spatial cell-cell interaction was determined with imcRtools (26) by constructing a spatial graph on expansion base with a threshold of 20. Interaction enrichment was assessed using permutation testing (iter = 1000; p < 0.05).

## Results

### The IMC panel enables visualization and identification of (immune) cell subsets, and reveals the cellular composition of MF tumors and plaques

**Figure 1:**
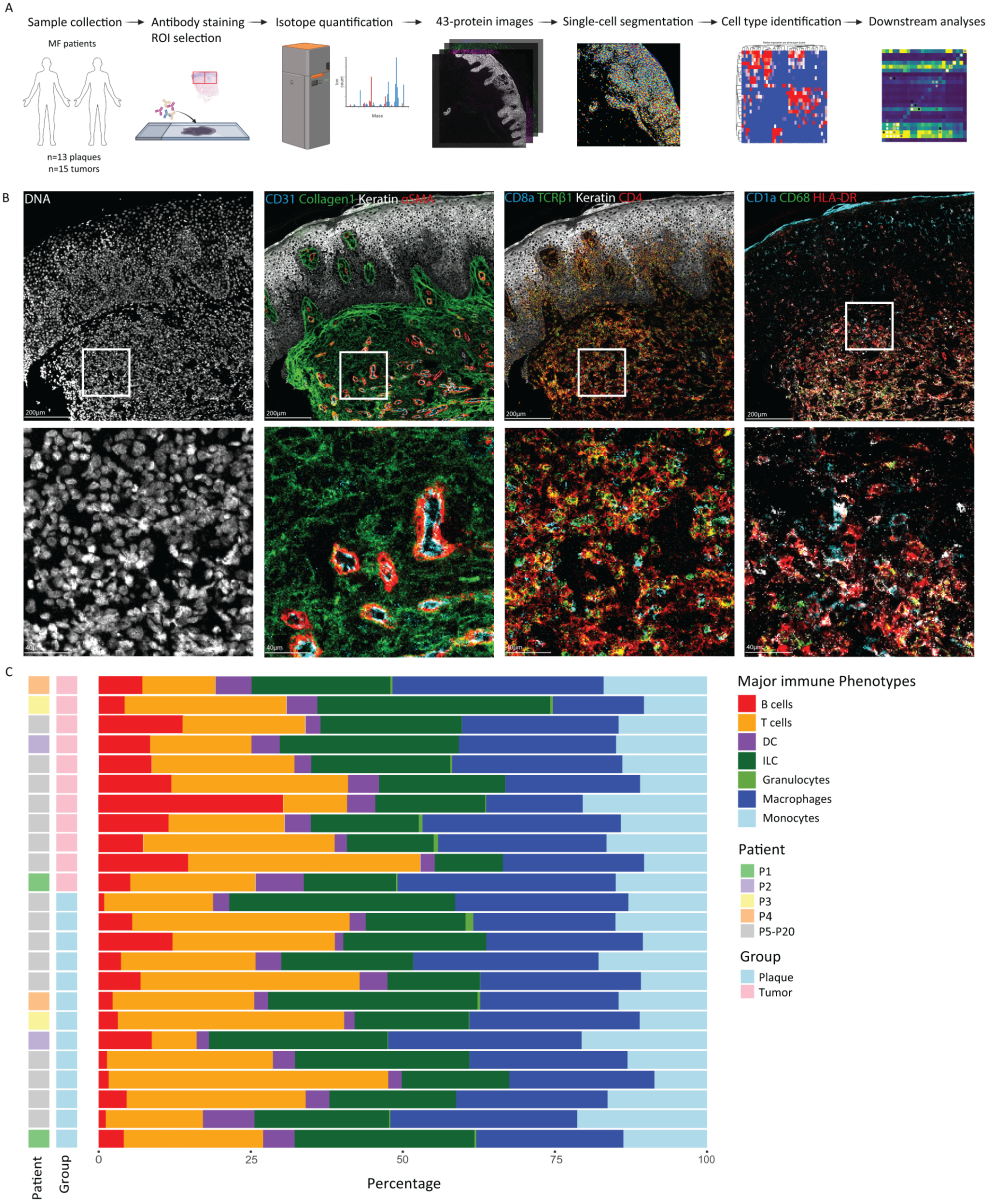
Representative images of IMC panel and immune cell compositions of MF tumors and plaques. **(A)** Workflow of IMC image acquisition from skin biopsies from MF patients, and downstream analyses. **(B)** IMC image reveals clear distinction of all immune cell lineages as well as structural organization of the tissue. Same Region Of Interest (ROI) is shown with different markers. From left to right: (1) DNA in white, (2) CD31 in cyanblue, Collagen1 in green, Keratin in white, alpha-SMA in red, (3) CD8a in cyanblue, TCRβ1 in green, Keratin in white, CD4 in red, (4) CD1a in cyanblue, CD68 in green, HLA-DR in red. **(C)** Percentage of major immune cell subsets is depicted, sorted per group (plaque/tumor) and patient sample.

To characterize and visualize immune cells in their spatial context in the tumor microenvironment of MF, we designed and optimized a 43-marker IMC panel for application to human skin samples (**Table 2**). Each marker is depicted in **Supplementary Figure 1**. The developed panel provides a thorough overview of the MF tumor microenvironment and specifically allows the visualization of structural markers (such as CD31, collagen, keratin, and α-SMA), various T cell subsets, and myeloid subsets (**Figure 1B**).

In this study, we subjected 27 skin biopsies of both plaques and tumors from patients with classical CD4+ MF to IMC using the newly developed panel. IMC requires extensive data analysis to segment cells and identify cell types within tissues. Data analyses included background removal, cell segmentation, and dimensionality reduction to cluster distinct phenotypes using marker expression profiles (**Figure 1A**). This resulted in the identification of 4 subsets of dendritic cells (DCs), 2 subsets of macrophages, 4 subsets of monocytes, 7 subsets of T cells and 5 structural subsets. Especially in the myeloid compartment, we were able to identify various subsets including CD11c+CD11b+ DCs, CD11c+CD11b- DCs, HLADR-CD14+ monocytes and HLADR+CD14+ monocytes. We identified 4 CD4+ T cell subsets including 2 tumor T cell subsets (TCRβ1+CD7-CD4+ T cells and CD30+TCRβ1+CD7-CD4+ T cells). Although it is known to be challenging to identify tumor cells in MF, we were able to identify two CD4+ T cell phenotypes that were most likely tumor cells, based on their spatial location and the expression of CD4, CD3, CD30, TCRβ1, CD7 and CD45. This was correlated with the morphology of the cells in consecutive HE staining (**Supplementary Figure 3**), and IHC stainings of CD3, CD4, CD5, CD7, CD8 and CD30. Tumor T cells express TCRβ1, CD4, CD3, and CD45. They often show a loss of CD7 and can express CD30. Tumor T cell expression can differ between patients, therefore the tumor T cell phenotypes were correlated to the sample. All identified cell types were counted and sorted into major cell subsets: B cells, T cells, innate lymphoid cells (ILCs), dendritic cells (DCs), granulocytes, macrophages, monocytes, keratinocytes, fibroblasts, and structural cells (**Supplementary Figure 2A**). The relative abundance (percentage) of major immune cell subsets is shown in **Figure 1C**. Overall, there were no major differences between plaques and tumors regarding the major cell subsets, including lymphocytes, myeloid cells, stromal, and structural cells (**Supplementary Figure 2B**). The composition of the cell subsets was heterogeneous across plaque and tumor samples. Visualization of the relative abundance of the major immune cell subsets highlighted more B cells present in the tumors relative to the plaques, while other major immune cell subsets were comparable between plaques and tumors (**Figure 1C**). In addition to examining the relative abundances of the major cell subsets, we conducted a detailed analysis of specific cell phenotypes to compare their absolute counts and relative percentages between plaques and tumors.

### A shift of cellular distribution in plaques and tumors

**Figure 2:**
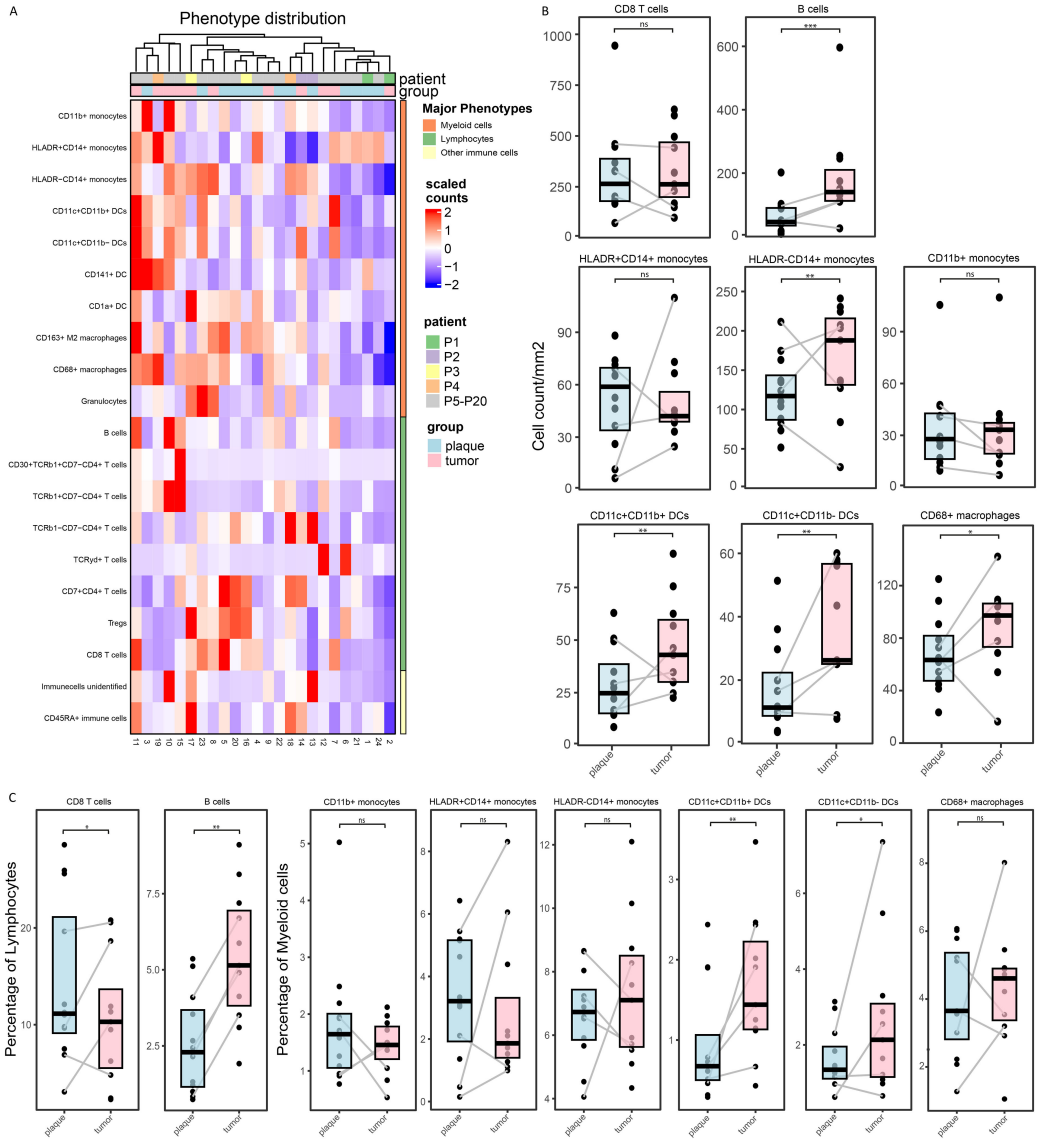
Phenotype distribution in plaques and tumor biopsies of MF patients. **(A)** Hierarchical clustered heatmap of distribution of cell counts of all immune phenotypic subsets throughout plaque and tumor samples. Heatmap is sorted based on major immune cell subsets (myeloid cells, lymphocytes, and other immune cells). **(B)** Distribution of cell counts in plaques and tumors of CD8+ T cells, B cells, CD11b+ monocytes, HLADR+CD14+ monocytes, HLADR-CD14+ monocytes, CD11c+CD11b+ DCs and CD11c+CD11b- DCs. **(C)** Distribution of cell percentages of B cells and CD8+ T cells of total percentage of lymphocytes. And distribution of cell percentages of CD11b+ monocytes, HLADR+CD14+ monocytes, HLADR-CD14+ monocytes, CD11c+CD11b+ DCs and CD11c+CD11b- DCs of total percentage of myeloid cells. Mann-Whitney U-test were performed. *p-value <0.05, **p-value <0.01, ***p-value <0.001.

In a more detailed approach, we focused on the distinct phenotypes and their distribution in plaques and tumors. First, we investigated the densities of all immune cell subsets. Hierarchical clustering of the heatmap did not reveal a clear separation between plaques and tumors (**Figure 2A**). The matched plaque and tumor biopsies from four patients did not cluster together except for patient 2 (**Figure 2A**). Furthermore, no consistent patterns in cell counts or percentages were observed across the matched samples (**Figures 2B, C**). Previous studies on the TME have primarily focused on CD8+ T cells, B cells, monocytes, and dendritic cells (13, 27–29). In our analysis, we examined these subsets in more detail to evaluate prior findings and add spatial insights. As previously reported in a study focusing on CD8+ T cells in MF (12), our analysis found no significant difference in CD8+ T cell counts between plaques and tumors. However, the percentage of CD8+ T cells among all lymphocytes was significantly higher in plaques than in tumors (p=0.04). In contrast, consistent with previous reports (13), B cells significantly increased in tumors, both in cell counts (p=0.0049) and as a percentage of total lymphocytes (p=0.0016) (**Figures 2B, C**), and as previously depicted in the distribution of major cell subsets (**Figure 1C**). In the myeloid compartment, the counts and percentages of CD11b+ monocytes and HLADR+CD14+ monocytes were comparable. HLADR-CD14+ monocyte cell counts increased significantly (p=0.008), while the percentage of myeloid cells were only slightly increased in the tumors compared to the plaques. In addition, CD11c+CD11b+ DCs and CD11c+CD11b- DCs were significantly higher in tumors than in plaques both regarding cell counts (p=0.0018, p=0.0034) and percentage of myeloid cells (p=0.039, p=0.009) (**Figures 2B, C**). CD68+ macrophage counts were significantly higher in tumors in comparison to plaques (p=0.0153), whereas percentages of myeloid cells were comparable (**Figures 2B, C**). CD163+ M2-like macrophage counts and percentages of myeloid cells were comparable between plaques and tumors (**Supplementary Figure 2C**). In conclusion, both in frequencies and relative proportions, tumors exhibited an increase in B cells, DCs, and monocytes, along with a reduction in the relative proportion of CD8+T cells, compared to plaques. This shift in cellular composition underscores the complexity of the TME and suggests that spatial organization may further distinguish tumors from plaques.

### Spatial analysis of cellular interactions reveals differences in tumor cell interactions between plaques and tumors

**Figure 3:**
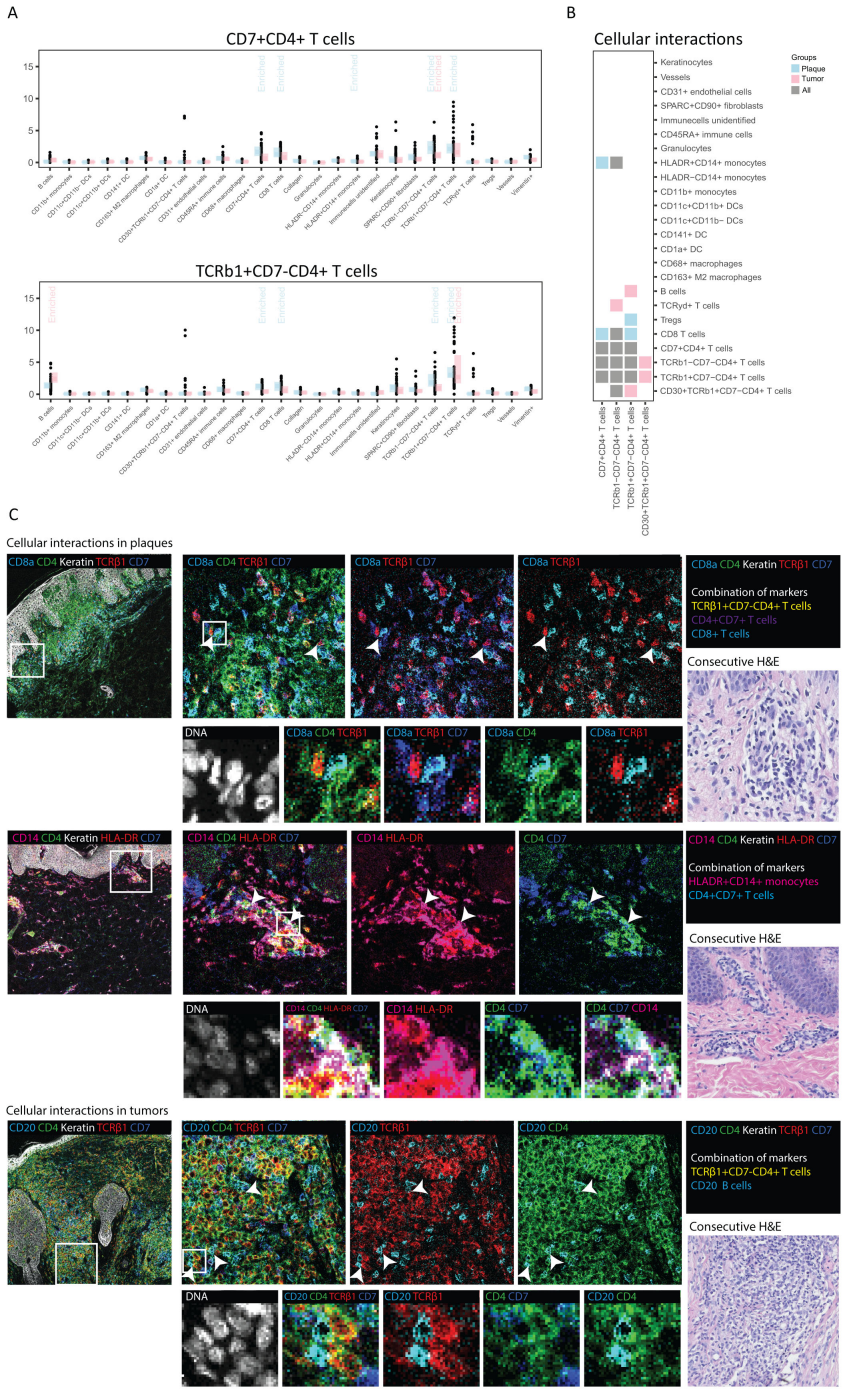
Cellular interactions in plaques and tumors. **(A)** The significantly enriched cellular interactions of the four CD4+ T cell subsets in tumors and plaques. The significantly enriched interaction between cell types occurs more frequently than random observation (p<0.05). Enriched in blue means significantly enriched interaction between said CD4+ T cell subset and interacting phenotypes in plaques. Enriched in pink means significantly enriched interaction between said CD4+ T cell subset and interacting phenotypes in tumors. **(B)** The significantly enriched cellular interactions of the four CD4+ T cell subsets compared between tumors and plaques. In gray, significantly enriched interactions that are present in both plaque and tumor. In pink, significantly enriched interactions only in tumors. In blue, significantly enriched interactions only in plaques. **(C)** IMC images of plaques and tumors confirming the cellular interactions between various phenotypes. Consecutive H&E images of corresponding IMC images.

In addition to differences in cell composition, variations in TME between plaques and tumors may also arise from differences in spatial organization, including cellular interactions and neighboring cell populations. To investigate cell-cell interactions, we determined whether the interaction between cell types occurred more frequently than the random distribution of cells. We investigated direct cell-cell neighbors and compared them between plaques and tumors. We observed significantly enriched interactions between cells of the same lineage (e.g. T cells and monocytes) in both plaques and tumors (**Supplementary Figures 2D, E**). Interestingly, if we look at the cellular interactions of the four CD4+ T cell subsets, there are several differences between the interacting cells in plaques and tumors (**Figures 3A, B**; **Supplementary Figure 2E**). We observed 4 notable significantly enriched cell-cell interactions that differed between plaques and tumors. First, in plaques, but not in tumors, cell-cell interactions occur between CD4+CD7+ T cells and CD8+ T cells. Second, in plaques, there are cell-cell interactions between TCRβ1+CD7-CD4+ Tumor T cells with CD8+ T cells and FoxP3+ Tregs. Furthermore, cell-cell interactions between HLADR+CD14+ monocytes and CD7+CD4+ T cells were significantly enriched in plaques but not in tumors. Finally and notably, not only were B cells more abundant but they also interacted with TCRβ1+CD7-CD4+ Tumor T cells in tumors, a pattern not observed in plaques (**Figure 3B**). These proximities were confirmed in IMC images (**Figure 3C**). Overall, we observed a shift from CD8+ T cell–tumor cell and monocyte–CD4+ T cell interactions in plaques to B cell–tumor cell interactions in tumors.

## Discussion

Applying a custom made IMC panel, we conducted a comprehensive spatial analysis of the tumor microenvironment (TME) across early and advanced stages of MF. Our data revealed stage-specific changes in the TME that may contribute to anti-tumor immune response or tumor progression in MF. Specifically, early-stage MF lesions contain higher proportions of active cytotoxic CD8+ T cells as a percentage of total lymphocytes, whereas advanced-stage lesions exhibit increased proportions of B cells and altered immune cell interactions that could promote tumor growth.

Although previous studies on the TME of MF have identified differences between plaques and tumors, the spatial organization of these cell types remains insufficiently understood. In our dataset, hierarchical clustering of cell counts did not clearly separate patches/plaques from tumors, suggesting that disease-stage differences arise not only from absolute cell counts, but also by shifts in relative proportions and cellular interactions. These findings underscore the importance of integrating spatial context with detailed phenotyping to capture the full complexity of the TME. Within our MF cohort, we identified distinct cellular populations that varied between plaques and tumors, including CD8+ T cells, B cells, monocytes, and dendritic cells.

Previously, the percentage of CD8+ T cells in total lymphocytes was shown to be decreased in tumor in comparison to plaques in MF (12). In line with these observations, we found that although there was no difference in the absolute counts of CD8+ T cells between tumors and plaques, the percentage of CD8+ T cells was significantly lower in tumors. The protective role of CD8+ T cells in preventing MF progression is a topic of speculation. CD8+ T cells are traditionally recognized for their cytotoxic function and protective role in cancers such as melanoma, colorectal cancer, and ovarian cancer (30–32). This observation is supported by *in vitro* studies in CTCL, which demonstrate that CD8+ T cells suppress CD4+ T-cell proliferation (33). By demonstrating the cellular interaction between CD8+ T cells and tumor cells in plaques but not in tumors, our data further support the notion that CD8+ T cells are functionally relevant in preventing the progression of early stages of MF. Given their cytotoxic potential to eliminate tumor cells, to be confirmed by functional tests, CD8+ T cells may also represent a promising focus for therapeutic stimulation in advanced MF.

Recent spatial transcriptomic analyses have revealed a higher abundance of B cells in MF tumors than in plaques, and their presence was correlated with poor prognosis. MF tumors have been further characterized by the expression of genes involved in T−cell activation, B−cell recruitment, and formation of B−cell aggregates (13). These B−cell aggregates have been implicated in disease progression by facilitating interactions between tumor cells, B cells, and stromal cells (13, 34). Consistent with these observations, we validated the significantly increased abundance of B cells in MF tumors compared to that in plaques. In addition, we identified significantly enriched cell–cell interactions between tumor cells and B cells in tumors, but not in plaques. This stage-specific enrichment is particularly notable, as it may indicate that B cell–mediated crosstalk becomes more prominent during disease progression and could contribute to the transition from early to advanced stages. B cells may facilitate these interactions by providing growth factors and mediating antigen presentation. Together, these findings support the view that B cells are an integral component of the MF tumor microenvironment and highlight their potential as therapeutic targets, including B-cell-directed approaches such as rituximab.

Growing evidence has highlighted the role of monocytes in shaping the immune response to cancer (35). Certain monocyte-derived cells in melanoma and intestinal tumors cross-present tumor antigens and activate CD8+ T cells (36–38). Furthermore, monocytes and dendritic cells have been shown to interact with T cells in early stage MF (29). While monocytes initially contribute to anti-tumor immunity in skin cancer, their role in the TME often shifts to tumor promotion (39), potentially supporting malignant T cell growth in advanced CTCL (38). In our study, HLADR-CD14+ monocytes, CD11c+CD11b+ DCs and CD11c+CD11b- DCs significantly increased in tumors. However, dendritic cells showed no relevant interactions with CD4+ T cells. HLADR+CD14+ monocytes interact with CD7+CD4+ T cells in plaques but not in tumors, suggesting that early-stage lesions retain active CD4^+^ T-cell engagement, whereas this interaction diminishes with tumor progression, potentially facilitating immune evasion. Furthermore, HLADR+CD14+ monocytes interacted with tumor T cells in both plaques and tumors. The role of this interaction could be dual-faceted, with potential pro- and anti-tumor effects (27). The balance between these opposing roles may depend on the tumor stage, cytokine milieu, and broader immune landscape, ultimately shaping the effectiveness of the anti-tumor response. Further investigation is needed to explore potential therapeutic strategies that can enhance monocyte-mediated anti-tumor immunity while preventing their immunosuppressive functions.

Although shifts in the relative abundance, frequency, and interactions of B cells, CD8+ T cells, and monocytes were observed across MF stages, the matched plaque and tumor biopsies from the four patients showed no consistent patterns. Additionally, plaques from later-stage MF did not exhibit differences compared to those from early-stage MF. This underscores a limitation of our study, highlighting the need for a larger patient cohort to identify potential patterns in matched plaque and tumor samples. Furthermore, functional studies are warranted to validate the cellular interactions observed and to define the functional states and detailed phenotypes of these abundant B cells, CD8+ T cells, and monocytes.

Overall, this study demonstrates the importance of spatially resolved analyses of detailed immune cell subsets at the protein level to understand stage-specific alterations in the tumor microenvironment (TME) of mycosis fungoides. Specifically, we identified an increased abundance of B cells alongside a decreased proportion of CD8^+^ T cells in advanced disease stages, and by including spatial cellular interactions, we reveal a shift from CD8^+^ T cell–tumor cell interactions and monocyte/CD4+ T cells interactions in plaques to B cell–tumor cell interactions in tumors. Together, these findings offer novel insights into stage-specific remodeling of the tumor microenvironment in mycosis fungoides and highlight potential avenues for therapeutic intervention.

## Data Availability

The raw data supporting the conclusions of this article will be made available by the authors, without undue reservation.

## Supplementary material

Supplementary Figure 1 **(A)** Images depicting one ROI of MF tumor biopsy. All markers positive in this sample of the CTCL IMC panel are shown. **(B)** Images depicting one ROI of MF tumor biopsy that is positive for the markers CD30, CD47, PD-1, PD-L1 and Granzyme B.

Supplementary Figure 2 Cell subsets identified in tumors and plaques and their cellular distribution and interactions. **(A)** Marker expression by identified cell subsets. **(B)** Distribution of cell subsets in plaques and tumors. **(C)** Distribution of cell counts in plaques and tumors of CD163+ M2 macrophages and distribution of cell percentages of CD163+ M2 macrophages of total percentage of myeloid cells. Mann-Whitney U-test were performed. * = p-value <0.05, ** = p-value <0.01, *** = p-value <0.001. **(D)** Cellular interaction plot for plaques and tumors, tested for significance. White dots indicate significantly enriched interactions (p<0.05). The significantly enriched interaction between cell types occurs more frequently than random observation.

Supplementary Figure 3 **(A)** Consecutive H&E image of two ROI of IMC images of two MF plaque biopsies, correlating to **Figure 3**. **(B)** Consecutive H&E image of one ROI of IMC images of one MF tumor biopsy, correlating to **Figure 3**.
