# Supplementary figures and images for: Cellular interaction of mycosis fungoides tumor cells changes from cytotoxic CD8+ T cells in plaques to B cells in tumors
Source: Front Immunol. 2026 Jul 2;17:1823626. doi: 10.3389/fimmu.2026.1823626 (PMC13372751; doi:10.3389/fimmu.2026.1823626)

A

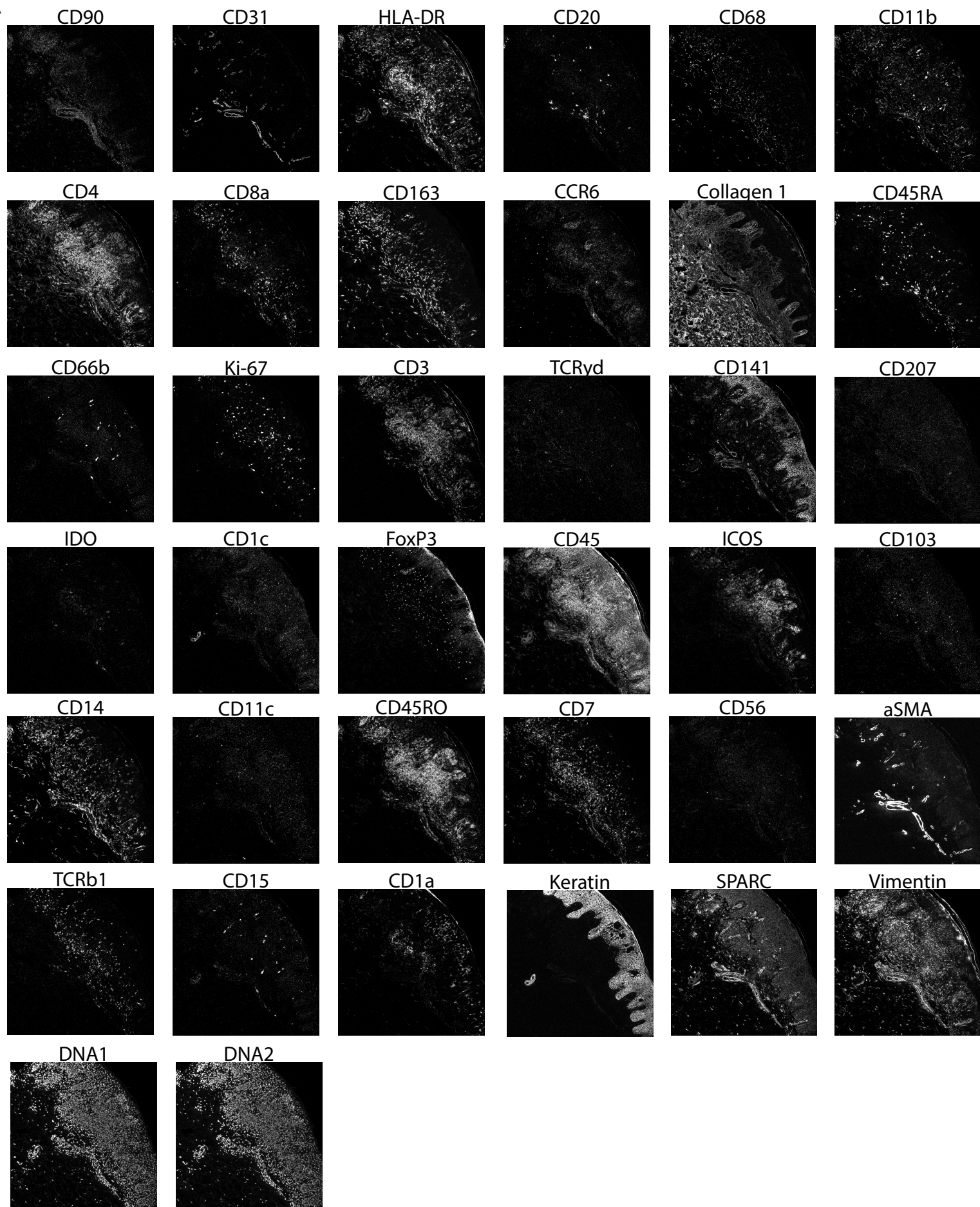

B

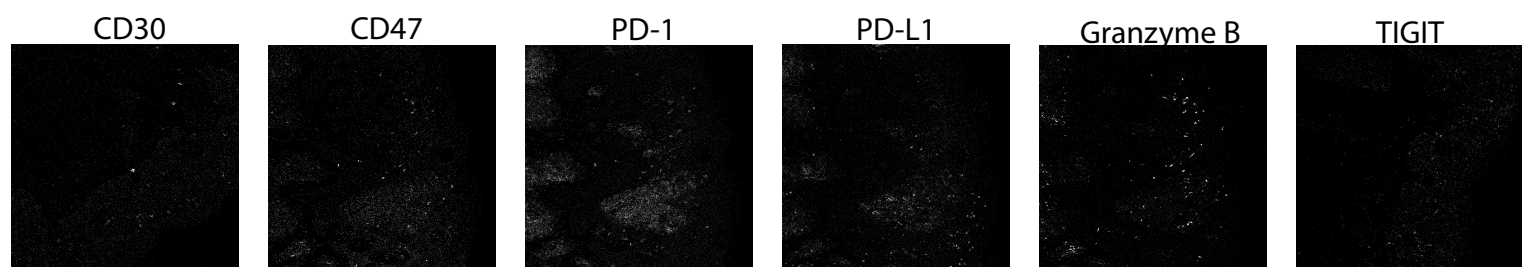

Supplement: Supplementary Figure 1 — (A) Images depicting one ROI of MF tumor biopsy. All markers positive in this sample of the CTCL IMC panel are shown. (B) Images depicting one ROI of MF tumor biopsy that is positive for the markers CD30, CD47, PD-1, PD-L1 and Granzyme B. [file DataSheet1.pdf]

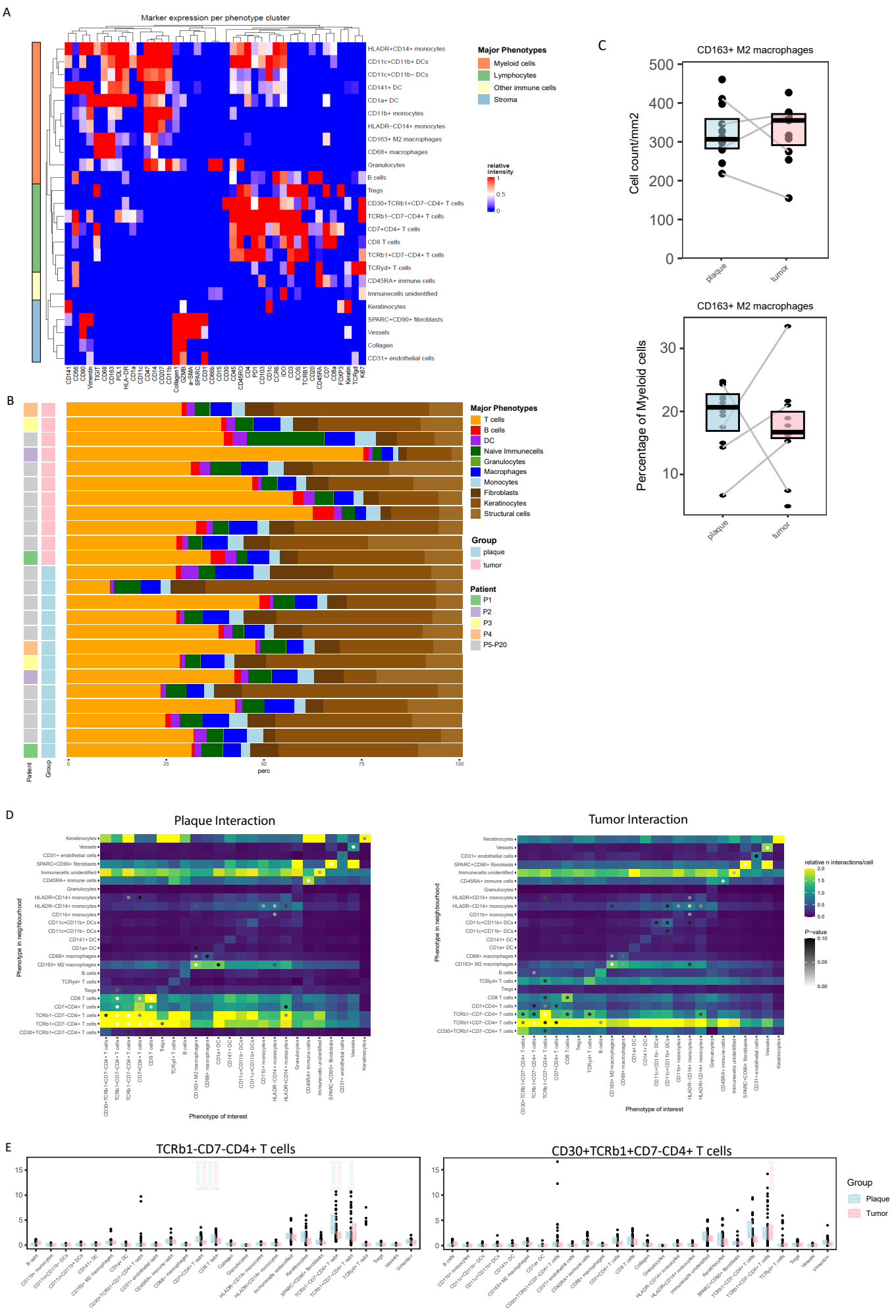

Supplement: Supplementary Figure 2 — Cell subsets identified in tumors and plaques and their cellular distribution and interactions. (A) Marker expression by identified cell subsets. (B) Distribution of cell subsets in plaques and tumors. (C) Distribution of cell counts in plaques and tumors of CD163+ M2 macrophages and distribution of cell percentages of CD163+ M2 macrophages of total percentage of myeloid cells. Mann-Whitney U-test were performed. * = p-value <0.05, ** = p-value <0.01, *** = p-value <0.001. (D) Cellular interaction plot for plaques and tumors, tested for significance. White dots indicate significantly enriched interactions (p<0.05). The significantly enriched interaction between cell types occurs more frequently than random observation. [file DataSheet2.pdf]

**A** Plaque samples

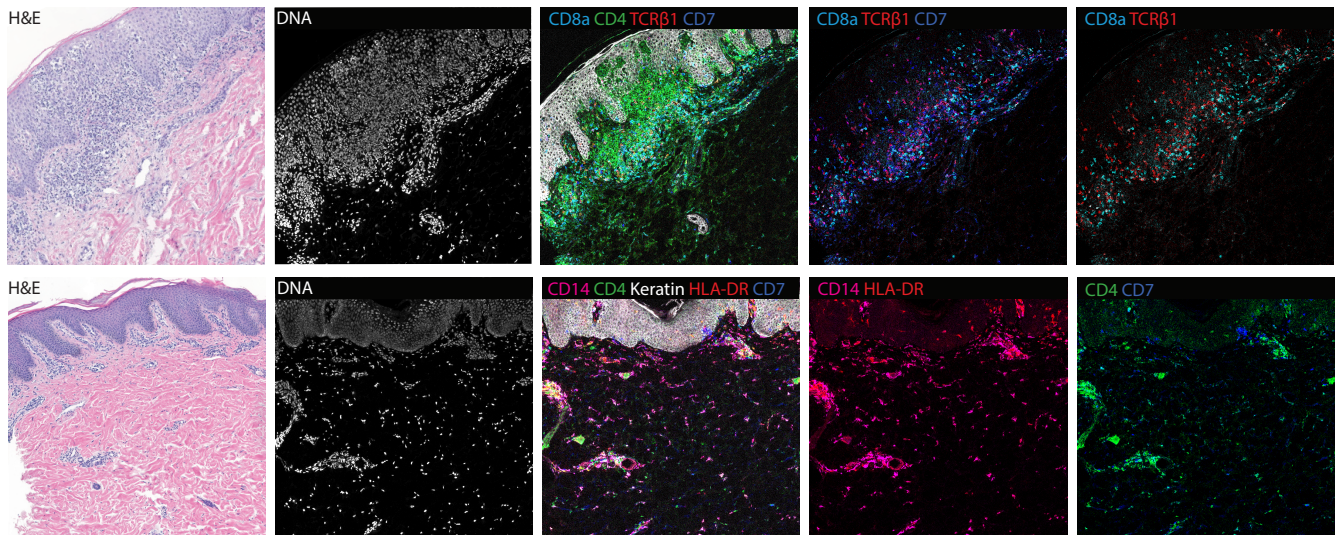

**B** Tumor samples

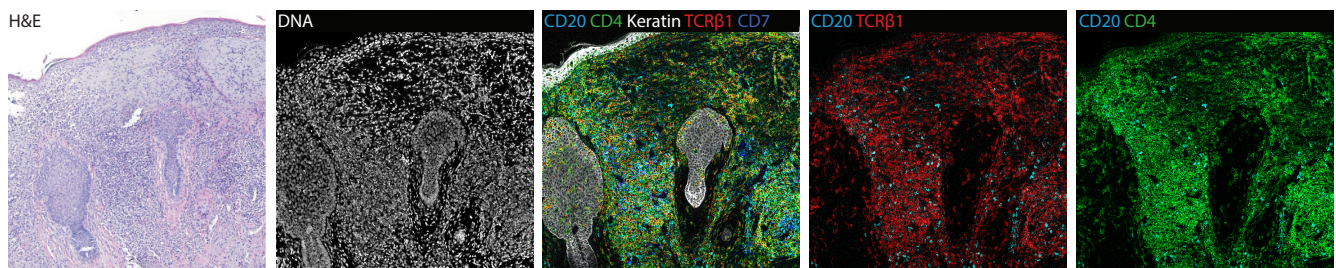

Supplement: Supplementary Figure 3 — (A) Consecutive H&E image of two ROI of IMC images of two MF plaque biopsies, correlating to Figure 3. (B) Consecutive H&E image of one ROI of IMC images of one MF tumor biopsy, correlating to Figure 3. [file DataSheet3.pdf]
